# The gut microbiome, immune check point inhibition and immune-related adverse events in non-small cell lung cancer

**DOI:** 10.1007/s10555-022-10039-1

**Published:** 2022-07-25

**Authors:** Philip Bredin, Jarushka Naidoo

**Affiliations:** 1 Beaumont RCSI Cancer Centre, Dublin, Ireland; 2 The Bloomberg-Kimmel Institute of Cancer Immunotherapy, Johns Hopkins University School of Medicine, Baltimore, MD USA; 3 Department of Oncology, Johns Hopkins University School of Medicine, Baltimore, MD USA

**Keywords:** Non-small cell lung cancer, Gut microbiome, Immune checkpoint inhibitor, Immune-related adverse events, Immunotherapy

## Abstract

Systemic treatment options for patients with lung cancer have expanded in recent years, with a number of immunotherapeutic strategies now in our treatment armamentarium. Toxicity of and resistance to treatment hold a major stake in lung cancer morbidity and mortality. Herein, we summarise the background, current evidence and potential mechanisms underlying the role of the commensal gut microbiota in immunotherapy outcomes such as response and toxicity in patients with non-small cell lung cancer (NSCLC).

## Introduction and terminology

A growing body of evidence has emerged linking a patient’s gut microbiome (GM) and their anti-cancer immune response in those treated with immunotherapy. Immune evasion, such as through expression of immune checkpoint proteins, is a hallmark of carcinogenesis. In the past decade, immune checkpoint inhibitors (ICI) have been profoundly successful in treating certain solid malignancies. Worse outcomes are observed in patients with non-small cell lung cancer (NSCLC) treated with antibiotics (ABX) prior to ICI. Given that ABX induce gut dysbiosis, the term for a disordered microbiome, this suggests a theoretical link between the GM and ICI activity, which could have major implications for patients’ cancer outcomes. In this review, we will first put this theory into context by familiarising the reader with common terminology and techniques used in studies of the GM, and by summarising the relevant history and mechanisms of ICI and immune-related adverse events in NSCLC. We will then discuss the current evidence on key issues in regard to the links between the GM and ICI outcomes in NSCLC, including the mechanism of the GM’s influence on the anti-cancer immune response, the strength of the evidence for negative ICI outcomes with use of ABX in NSCLC, the characteristics of the GM that may lead to positive or negative ICI outcomes, including response and toxicity, and the manipulation of the GM for therapeutic purposes.

### The microbiome and microbiota: roles in host immunity

The microbiome refers to the collective genomes of the species in a particular biological niche, which in humans includes microbiota such as bacteria, fungi and viruses, which live commensally or symbiotically with the human body [1]. The local microbiome varies by organ such as the gut, skin, mouth and airways. The human microbiome has evolved to form part of the innate immune system [2]; for example, the absence of commensal skin flora with an enzyme degrading *Staphylococcus* leads to atopic dermatitis [3]. The relationship between the GM and the immune system is bidirectional: The GM contents are regulated by the local immune system of the gut, and in turn contribute to the activity of the local and systemic immune systems [4]. In the adaptive immune system, tolerance is required of commensal organisms in order to prevent systemic inflammation [5]. Interactions between the immune system and the commensal microbiota can determine systemic immune tone, including surveillance of malignant cells [6].

**Fig. 1:**
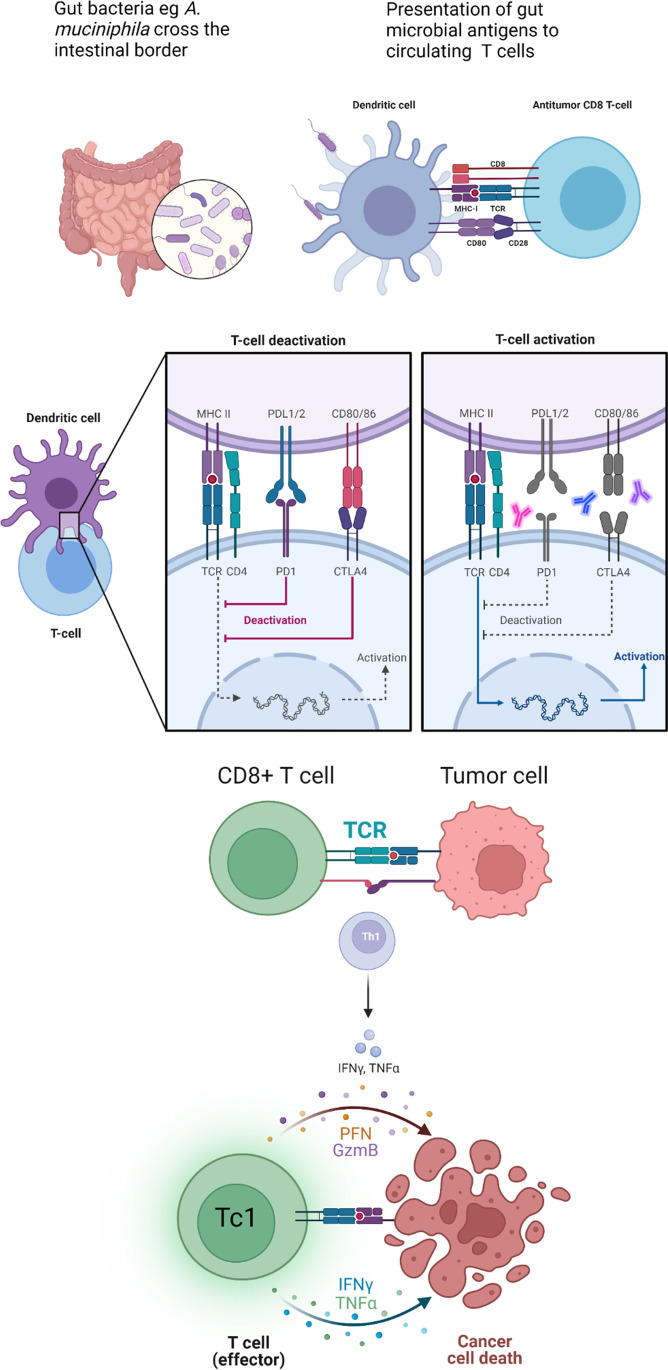
The role of the gut microbiome in immune checkpoint inhibitor response and toxicity: Commensal gut bacteria such as *Akkermansia muciniphila* cross the intestinal border and interact with antigen-presenting cells, such as dendritic cells, in the local immune microenvironment. Antigens of gut microbes are continuously presented to circulating CD8 cytotoxic T lymphocytes involved in antitumor immune surveillance, which are primed for tumour killing due to overlap between tumour and gut microbe antigens. Tumour cells express immune checkpoints such as PD-1 and CTLA-4 in order to evade this immune response. Introduction of immune checkpoint inhibitor(s) allows for upregulation of T cell activation, leading to release of cytokines involved in cancer cell death including IFNγ, TNFα, PGN and GzmB. Glossary: MHC, major histocompatibility complex; TCR, T cell receptor; PD-L1/2, programmed cell death ligand 1/2; PD-1, programmed cell death protein-1; CTLA-4, cytotoxic T lymphocyte-associated protein 4; Th1, helper T lymphocyte 1; PFN, perforin; GzmB, granzyme B; Tc1, cytotoxic T lymphocyte 1; IFNγ, interferon gamma; TNFα, tumour necrosis factor alpha. Created with BioRender.com

The GM is composed largely of anaerobic bacteria [7] which function to aid digestion. The immune microenvironment at the border of the intestine develops tolerance for the presence of these bacteria thereby not recognising them as pathogens [8]. Translocation of gut microbiota to the local lymphoid organs facilitates the systemic immunomodulatory activity of the gut microbiota (see Fig. 1).

### Dysbiosis

Dysbiosis, the term for an abnormal microbiome, has long been associated with autoimmune conditions such as inflammatory bowel disease [9]. Dysbiosis can directly lead to disease from pathogens such as *Clostridioides difficile* [10]. ABX are a common cause of dysbiosis [11, 12]. Dysbiosis can also exist in disease states in the absence of antibiotic administration, and may be related to a number of host factors such as age, diet, environment, co-morbidities and other medications including proton pump inhibitors [13, 14]. The degree of dysbiosis with ABX may vary with the dose, duration and antimicrobial spectrum of the antibiotic used, as well as the baseline GM of the patient. Standardising the study of dysbiosis is difficult due to its complex and dynamic nature, and there may be little overlap between descriptive studies of the GM in similar populations with small sample sizes.

### Microbial diversity

Studies of the GM may identify the presence of specific organisms associated with particular treatment outcomes, including the relative abundance of these organisms; however, other features of the GM which may be influential can also be measured. Alpha diversity, often described in terms of the Shannon diversity index, refers to the richness of the full spectrum of organisms present within a particular sample or community, both in terms of the number of different organisms present, and the abundance of those organisms. It has been suggested that alpha diversity, as a summative measure of the contents of the GM, may correlate better with cancer treatment outcomes than the abundance or absence of a particular microorganism [15]; however, this link has not yet been proven decisively and it is likely that both factors are important. Beta diversity refers to the difference between communities or samples, and is less frequently referred to in the studies described in this article.

### Methods to characterise the GM

Traditional microbial culture techniques are subject to the organisms’ growth requirements which are prohibitive in the majority of cases for commensal gut flora. Novel techniques of sequencing genes in large scales and of multiple organisms simultaneously have allowed for descriptions of the composition of the GM at a metagenomic level [16–18]. The process of classification and cataloguing of all microorganisms detected using these methods is ongoing. Nomenclature of previously described microorganisms may change as new data emerges. The summative microbial contents of the gut can be analysed by subjecting stool samples from individual patients to gene sequencing. The two laboratory methods most frequently used to achieve this are 16S ribosomal ribonucleic acid (rRNA) sequencing and shotgun metagenomic sequencing.

The 16S rRNA gene is unique to bacteria, and sequencing of this gene in whole or in part has been used for several decades to differentiate between bacterial species. Differences in the less-conserved parts of the 16S rRNA gene are used to identify different taxa. Reference to databases such as Greengenes [19] is required to identify known and novel taxa. Search algorithms such as the National Cancer Institute’s Basic Local Alignment Search Tool (BLAST) are specifically designed for this purpose, and continue to be augmented as larger and more complex datasets are generated [20]. Similar gene sequences may be clustered into operational taxonomic units (OTUs) and otherwise unclassified gene clusters are named to the lowest possible taxonomic level, such as uncultured genus-level group (UCG). Modern techniques allow for rapid sequencing of the whole 16S rRNA gene, which gives more specificity than older methods which focused on individual segments [21]. Bioinformatics software, such as the computational tool Resphera [22], is used to analyse the large amounts of data which are generated. This is required in order to filter out noise and sort the information into the clinically relevant datapoints. 16S rRNA sequencing is limited to identifying bacteria at a species level, and differentiating between all clinically relevant strains may not be achievable with this method due to technical limitations [23]. The majority of 16S data will only get to genus level, and species level identification is dependent on either sequencing multiple regions of the 16S gene, which is not yet commonly performed, and/or on bioinformatic tools, as well as the bacterial population being studied.

Metagenomics refers to the genome of a whole sample population, which in the case of the GM includes genetic material from multiple organisms. Shotgun metagenomic sequencing is a type of whole-genome sequencing which analyzes all of the genomic material of a stool sample, including non-bacterial organisms and human cell DNA. It therefore gives much more granular detail than 16S rRNA sequencing, but is associated with higher cost and can be more resource-intensive [24]. Differentiating between bacterial strains, and functional profiling such as measuring antibiotic resistance, is possible with metagenomic sequencing [25]. Gene sequences from different organisms are detected in a random or “shotgun” manner and assembled into contiguous sequences before identifying the taxonomy of the microbiota present using reference databases such as MG-RAST [26]. Many computational tools are available to carry out this classification, and often perform multiple functions such as cleaning and filtering the data prior to classification, such as the pipelines offered by bioBakery 3 [27].

Both shotgun metagenomic sequencing and 16S rRNA sequencing give detailed descriptions of the GM and can be used separately or in combination depending on the goal of the study. The majority of clinical studies we will describe here used one method in order to identify the taxa of the organisms correlating with outcomes, as well as the alpha diversity of the samples.

### The GM and cancer therapeutics: past and future

Before the era of ICI, the GM was shown to influence outcomes of cytotoxic chemotherapy via modulation of the immunological response to gut bacteria. For example, cyclophosphamide, when studied in a pre-clinical model, induces translocation of immunogenic bacteria such as *Enterococcus hirae* across the gut wall to lymphoid organs. This in turn stimulates production of antitumor T lymphocytes [28, 29] via mechanisms such as molecular mimicry. Further clinical studies have confirmed the interaction between chemotherapy and the GM, with a favourable GM at baseline leading to superior outcomes in multiple tumour types [30], including lung cancer [31]. On the other hand, chemotherapy can induce dysbiosis, which is associated with higher rates of adverse events [30].

The shifting of an unfavourable GM, antibiotic-associated or otherwise, to a favourable microbiota is the natural progression of the dysbiosis theory for therapeutic purposes. Methods to achieve this may include but are not limited to administration of probiotics, diet and prebiotics, or faecal microbiota transplant (FMT) from healthy subjects or those with better outcomes from a similar cancer treatment [32].

## Immune checkpoint inhibition in lung cancer

### Monoclonal antibodies to CTLA-4 and PD-1/PD-L1

The GM appears to modify both the natural history of solid cancers through its immunological sequelae, as well as cancer treatment outcomes via its interaction with immunotherapeutics, in particular with ICI. This may be explained through understanding the mechanisms of ICI. The immune checkpoint molecules cytotoxic T lymphocyte–associated protein 4 (CTLA-4) and programmed cell death protein–1 (PD-1) exist to carry out an essential function in immunological homeostasis. The ligands, cluster of differentiation (CD) 80 and CD86, and programmed cell death ligand 1 and 2 (PD-L1 and PD-L2) respectively, are co-expressed on cell surfaces with major histocompatibility complex (MHC) class I (Fig. 1). This functions to prevent an excessive or prolonged stimulation of the T cell receptor by antigens expressed by MHC class I, such as those from the GM, thus preventing the harm associated with chronic inflammation, and reducing the risk of autoimmunity. In the immunoediting process of carcinogenesis, tumour cells express immune checkpoint proteins in order to suppress the immune response against these cells. In the setting of an intact immune system, innate and adaptive immune reactions to eliminate tumours result in pressurised outgrowth of cells which have resistance to immune attack [33]. This results in equilibrium, where tumour cells survive in a dormant state, and eventually tumour proliferation via immune escape.

Inhibition of CTLA-4 and/or PD-1 and its ligand PD-L1 with ICIs can subject cancer cells to immune surveillance and immunogenic cell death via multiple mechanisms. These may include direct cytotoxic T cell-induced apoptosis and perforin-mediated lysis (Fig. 1), as well as antibody-dependent cellular cytotoxicity [34]. This strategy has changed the therapeutic landscape and significantly improved outcomes for patients with non-small cell lung cancer (NSCLC) based on several practice-changing phase III studies of both single agent [35, 36] and combination ICI approaches [37–41], in advanced and locally advanced [42] NSCLC. Measurement and/or modulation of the effects of the GM on ICI outcomes was not incorporated into these early landmark trials of ICI in NSCLC and therefore remains investigational, but is being increasingly incorporated into modern randomised controlled trials of these agents.

### Resistance to ICI

A major challenge to date has been primary and acquired resistance to ICI [43], identifying a potential role for the GM as a predictive biomarker and therapeutic target. Given the unprecedented rate of sustained responses to ICI in metastatic disease not previously achieved by systemic treatments such as cytotoxic chemotherapy, the emphasis on overcoming ICI resistance is a major area of interest in oncology. In the landmark trials of single-agent PD-1 inhibitors for NSCLC, and combined CTLA-4 and PD-1 inhibitors, the rate of primary progression was 27–44% [35, 44–50] and 23% [40] respectively. Sustained response to ICI is seen in 13–32% of patients with metastatic disease [51–53] but unfortunately the majority of patients with NSCLC develop acquired resistance to ICI, after initial disease response or stabilisation. Many predictive biomarkers for response to ICI have been proposed, such as PD-L1, which was validated based on KEYNOTE 010 [47] and is used in clinical practice, and tumour mutational burden (TMB) [54, 55] which has been studied widely but is not yet validated; however, these biomarkers are limited by issues related to interpretation. This means that neither PD-L1 nor TMB are both necessary nor sufficient to select for ICI therapy alone, and that patients whose tumours are either PD-L1 or TMB low may still respond to therapy. Resistance to ICI can be due to tumour-intrinsic factors of NSCLC, as well as tumour-extrinsic factors such as immune tone. An individual’s immune system in general has a set point of immune tone, influenced by a complex combination of tumour, host and environmental factors, that determine the strength and timing of the immune response to a tumour [6]. The host immune response must maintain a balance between pro-inflammatory states during antigen exposure, and regulation of immune responses, which cause harm if unchecked. Immune tone may be influenced by genetics as well as environmental factors, including alterations in the GM. Other predictive biomarkers have been proposed, including CD8 + T cell density, interferon-γ (IFN-γ)-related mRNA profile, the expression of MHC class I molecules on immune cells of the tumour microenvironment and/or the combination of a number of these [56], and further study is needed to define the clinical relevance of these biomarkers.

Resistance to ICI in NSCLC is evidently a major area of unmet need in terms of predictive markers as well as therapeutics. The reason for differences in response to ICI between individuals is unclear and it has been theorised that variations in the GM between individuals may in whole or in part account for resistance to ICI. The GM therefore holds promise as a basis for a predictive biomarker and may be favourable to the above-mentioned biomarkers given its potential as an additional therapeutic target to augment ICI activity and reduce ICI toxicity.

### Immune-related adverse events secondary to ICI

Immune-related adverse events (irAEs) are a specific class of toxicities that occur from ICIs that are related to the immunologic mode of action of these agents [57–59], and therefore may be influenced by the GM. The mechanisms involved in the pathophysiology of irAEs may include but are not limited to (1) T cell-mediated toxicity against shared antigens between tumour and irAE organ, (2) auto-antibody formation and (3) production of pro-inflammatory cytokines [57]. In certain cases, a direct effect of the monoclonal antibody against the cell is possible, with one example being hypophysitis caused by CTLA-4 expression on pituitary cells resulting in direct targeting by drugs such as ipilimumab [60, 61], but this is thought to be an exceptional phenomenon. Genetic predispositions to certain irAEs have been observed with variants of the human leukocyte antigen (HLA) gene, such as HLA-DRB1*11:01 and pruritus (odds ratio [OR] = 4.53, *p* < 0.01) as well as HLA-DQB1*03:01 and colitis (OR = 3.94, *p* = 0.017). The role of B lymphocytes in development of irAEs has been deduced from clinical studies: Patients on B cell-depleting therapies had zero cases of thyroiditis amongst 29 patients treated with pidilizumab, a PD-1 inhibitor, for follicular lymphoma [62], whereas this irAE typically occurs in 6–10% of patients with intact B cells. The pro-inflammatory cytokine interleukin-17 (IL-17), produced by T helper 17 (Th17) lymphocytes, appear to have a role in the development of ICI-induced colitis. Tarhini et al. measured circulating serum cytokines in 33 patients with locally advanced or metastatic melanoma treated with high-dose ipilimumab (10 mg/kg intravenously every 2 weeks) [63]. Higher baseline serum IL-17 levels were associated with significantly higher incidence rates of grade 3 colitis (*p* = 0.02). High doses of ipilimumab for metastatic melanoma have been shown to produce antibodies to enteric flora [64], which explains one potential mechanism for the high frequency of colitis with this agent. This theory may be extrapolated to explain the irAEs that occur at other sites that are exposed to commensal organisms, such as pneumonitis and skin toxicity.

In general, irAEs are graded by severity as follows: grade 1 = mild or asymptomatic; grade 2 = moderate or symptomatic requiring intervention or impairing activities of daily living; grade 3 = severe and/or requiring hospitalisation; grade 4 = life-threatening; and grade 5 = death. IrAEs occur in a large number of patients treated with ICI, with higher rates in patients who receive combination PD-1/CTLA-4 inhibition [39, 40, 65], CTLA-4 inhibition [66, 67] and anti-PD-1/PD-L1 monotherapy [35, 36, 38, 41, 47, 68] in that order. The majority of irAEs are mild, and manageable with observation, supportive care, and corticosteroids to reduce inflammation of the involved tissue or organ, and/or replacement of the relevant hormone in the case of endocrinopathies [58]. Hospitalisation or life-threatening toxicities occur in up to 10% of patients receiving ICI, and require higher doses + / − intravenous corticosteroids or steroid-sparing agents. The toxicities of immunosuppression for irAEs may contribute to the morbidity of ICI treatment. Immune-related adverse events of high grades are a rate-limiting factor for continuation of immunotherapy.

Response to ICI can persist post-cessation of ICI after an irAE [69, 70], but this phenomenon is not consistent or predictable. The relationship between irAEs and response to immunotherapy in NSCLC is controversial: irAEs are associated with improved PFS and OS across multiple studies in NSCLC treated with ICIs [71–73]. A multicentre retrospective analysis of 1010 patients treated with single-agent pembrolizumab in the first line for advanced NSCLC [70] found correlations between any site irAEs and objective response (*p* < 0.0001), PFS (*p* < 0.0001) and OS (*p* < 0.0001). Gastrointestinal irAEs were associated with better PFS (*p* = 0.0391). However, there are conflicting reports with regard to these associations, immortal time bias and attribution to ICIs [74].

### The advancing role of ICI in NSCLC

In NSCLC where the combination of chemotherapy with immunotherapy [37] is administered, the interaction between disease-drug-microbiome and associations with response or toxicity become more complex. Moreover, ICI in NSCLC has recently moved forward to the consolidation arena post-definitive radiotherapy [42], as well as neoadjuvant treatment [65, 75–77], asking more of the activity of ICI and allowing for new ways to study mechanisms of interaction, such as the effect of dysbiosis on pathologic response rates in surgical specimens [65].

## Mechanism of GM modulation of ICI activity

The mechanism of modulation of ICI response and toxicity by the GM has not been fully defined. There are multiple dynamic factors involved including the GM, the patient’s immune system and the characteristics of the NSCLC. Each of these is potentially modifiable, and interactions between them can change over time, leading to many permutations for when the ICI is added in. In the absence of ICI, through its interactions with the adaptive immune system, the GM is essential for anti-cancer immunity at baseline [4].

There are several purported steps in how the GM may prime immune responses to tumours in the context of ICI treatment. Firstly, there is a continuous exposure at the intestinal border of microbial antigens to host antigen-presenting cells (APCs). Immune tolerance of most GM antigens has developed to prevent harmful inflammation, but ongoing microbial antigen exposure to APCs is required in order for the adaptive immune system to remain primed for systemic tumour surveillance [78].

Secondly, individual microorganisms appear to translocate to local lymphoid organs and stimulate production of effector T lymphocytes which are directly active against cancer cells [28] (Fig. 1). Organisms such as *Akkermansia muciniphila* and *Enterococcus hirae* can stimulate helper T lymphocytes (Th1) and cytotoxic T lymphocyte (Tc1) cells, increasing release of cytokines such as IFN-γ [78]. Further downstream in this pathway, myeloid-derived suppressor cell activity within the tumour microenvironment can be inhibited [79].

Thirdly, when the ICI is introduced, the blockade of immune checkpoints appears to amplify the immune response to tumour antigens, which likely overlap with antigens from the GM [80]. A build-up of CXCR3 + CD4 + T cells in the tumour microenvironment as well as in the mesenteric and tumour-draining lymph nodes [78] is seen when a favourable GM is present at baseline prior to ICI.

Finally, the content of the GM microenvironment in terms of enzymes, cytokines and other molecules such as short-chain fatty acids [81], the “metabolome”, is influenced by the composition of the GM, and changes in this milieu may also alter ICI activity [82]; however, this is poorly understood.

## ICI and antibiotics in NSCLC

Many patients with NSCLC require antibiotic therapy for intercurrent infection. Since the introduction of ICI as standard of care for late-stage NSCLC in the early 2010s, the question of whether ABX lead to detrimental outcomes of ICI treatment has been posed. Arguments for and against this question, and evidence on variables such as timing of ABX, are described here.

Clinical studies are limited to retrospective and exploratory analyses for logistical reasons. A 2020 meta-analysis [83] concluded that the use of ABX within 60 days before or after commencing ICI was associated with a 6-month reduction in median overall survival (OS) in patients with NSCLC, with a pooled hazard ratio for OS of 1.69 (95% confidence interval [CI] 1.25–2.29). The between-study heterogeneity was high, and the authors noted that further work is required to establish whether this is a causal association.

The question of causation to date has been limited by confounding. Administration of ABX soon before or soon after commencement of ICIs has been associated with worse outcomes in multiple cancer types [84]. In general, risk factors for the development of NSCLC such as advanced age and smoking history are also risk factors for infection. Co-morbidities that predispose to infections, such as chronic obstructive pulmonary disease, are common in patients with NSCLC. Adverse features of lung cancer such as bronchial obstruction or neurological dysfunction from CNS involvement with aspiration can lead to lung infections. These confounding factors make a causal link between ABX and adverse immunotherapy outcomes more difficult to establish and may explain the inconsistency seen when attempts are made to reproduce this finding (see Table 1). Oral or intravenous ABX are a known cause of dysbiosis. Given what is known about the relationship between the GM and the immune system, many attempts have been made to prove a link between ABX, dysbiosis, and changes in efficacy of ICIs, and the presence and severity of irAEs.

**Table 1:** Findings from published peer-reviewed articles on the difference in overall survival (with medians and/or hazard ratio reported) for patients on immunotherapy with NSCLC treated with antibiotics near commencement of immune checkpoint inhibitors vs. those without antibiotic exposure in a given timeframe

First author	Year	Geographic location	Patients treated with ICI (*n*)	ABX (*n*)	mOS, ABX vs. no-ABX (months)	HR for death (95% CI)
Derosa [85]	2022	France and Canada	338	69 (20.4%)	NR	1.36 (0.95–1.94)
Verschueren [86]	2021	Netherlands	221	35 (15.8%)	NR	1.20 (0.79–1.85)
Ochi [87]	2021	Japan	531	98 (18.5%)	11.7 vs. 16.1	1.30 (0.92–1.85)
Giordan [88]	2021	France	65	18 (27.7%)	NR	2.11 (1.37–3.26)
Geum [89]	2021	Republic of Korea	140	70 (50%)	NR	2.29 (1.16–4.51)
Cortellini [90]	2021	International multicentre	302	47 (15.6%)	11.2 vs. 16.6	1.63 (0.99–2.68)
Cortellini [91]	2021	Europe	950	131 (13.8%)	5.6 vs. 19.3	1.42 (1.13–1.79)
Castello [92]	2021	Italy	50	20 (40%)	11.3 vs. 15.3	1.6 (0.7–53.7)
Chalabi [93]	2020	International multicentre	757	169 (22.3%)	8.5 vs. 14.1	1.32 (1.06–1.63)
Zhao [94]	2019	China	109	20 (18.3%)	NR	2.86* (1.3*-6.25*)
Pinato [95]	2019	England	119	6 (5.9%)	2.5 vs. 26	9.3 (4.3–19.0)
Ouaknine Krief [96]	2019	France	72	30 (41.7%)	5.1 vs. 13.4	2.2 (1.1–4.8)
Huemer [97]	2019	Austria	142	62 (43.7%)	14.6 vs. 11.2	0.91 (0.57–1.45)
Hakozaki [98]	2019	Japan	90	13	8.8 vs. nr	2.02 (0.70–5.83)
Galli [99]	2019	Italy	157	27 (17.2%)	5.9 vs. 11.9	1.064 (1.03–1.1)
Routy [78]	2018	USA	140	37 (26.4%)	8.3 vs. 15.3	2.21 (1.30; 3.74)
Derosa, Hellmann [100]	2018	USA	239	48 (20%)	7.9 vs. 24.6	4.4 (2.6–7.7)

*ICI* immune checkpoint inhibitors; *ABX* received antibiotics; *mOS* median overall survival; *HR* hazard ratio; *NR* not reported; *nr* not reached; *USA* United States of America

*Reported inversely

Routy et al. [78] identified strong links between the GM and outcomes in patients treated with PD-1 inhibitors in two large cancer centres in the USA and Europe. In their manuscript, Routy’s group describe an experiment with mouse models which were treated with broad spectrum ABX in order to deplete the gut microbiota prior to administration of ICI. Solid tumour models in these ABX-treated mice had poor responses to ICI compared to control mice with an intact GM. This generated the hypothesis that ABX-related dysbiosis at baseline can lead to worse response rates to ICI. This group simultaneously analysed 140 patients with advanced NSCLC treated with monoclonal antibodies to PD-1/PD-L1 in the second line or beyond. Similar baseline characteristics were seen when divided into groups of ABX-treated patients and those who did not receive ABX within 60 days prior to or 30 days after commencement of ICI. There was a clear difference between the groups’ outcomes, with a median survival of 8.3 months in the ABX-treated group vs. 15.3 months in the no-ABX group (*p* = 0.001). This finding was replicated in a validation cohort of 239 patients from the USA with median OS of 9.8 months vs. 21.9 months (*p* = 0.002) in the ABX vs. no-ABX groups. This was one of the first and largest studies to demonstrate a clear pre-clinical correlation between ABX exposure and worse outcomes from ICI treatment in solid tumours, and to match these findings in the clinical setting.

Derosa, Hellmann et al. [100] added to the work of Routy on the same validation cohort of 239 patients with NSCLC. One fifth of this cohort had exposure to ABX up to 30 days prior to commencement of ICI. The group of patients exposed to ABX within 30 days of ICI start again had significantly worse OS compared to the no-ABX group. In this study, a multivariate analysis was conducted to account for baseline factors that associate with poorer OS such as advanced age, prior lines of therapy and Eastern Cooperative Oncology Group (ECOG) performance status [100]. A marked difference in median OS of 24.6 months in the no-ABX group (*n* = 191) vs. 7.9 months (*p* < 0.01) for the ABX group (*n* = 48) and a HR of OS of 2.5 (95% CI 1.6–3.7, *p* < 0.01) was demonstrated. A shorter timeframe in the definition of ABX exposure appeared to show a greater difference in outcomes between the groups when compared to the results published by Routy et al. noted above.

These findings were replicated with more modest differences between groups in a large cohort of 3364 US armed forces veterans with NSCLC as presented at the American Society of Clinical Oncology (ASCO) Annual Meeting 2021 [101]. In this study, patients who received ABX within 30 days prior to ICI had a lower median OS of 7 months versus 10 months in those without prior ABX (*p* < 0.01). Receipt of ABX within 30 days prior to ICI was also associated with lower OS on multivariate analysis with a HR for OS of 1.31 (*p* < 0.01). This large sample size appears to show a smaller difference in outcomes between those with and without ABX exposure when compared to Routy and Derosa’s findings, with similar inclusion criteria.

The question of timing of ABX has been assessed in other ICI-sensitive tumours including melanoma. Mohiuddin et al. recently reported on a large database of patients with stage III and IV melanoma in which one fifth of the 568 patients received ABX within 3 months prior to ICI [102]. The hazard ratio (HR) for death ranged from 1.81 to 4.84 with ABX exposure. This association was seen across the board in subgroups based on the melanoma stage and whether the ICI was given in the adjuvant setting or for metastatic disease. This longer timeframe may represent prolonged dysbiosis after antibiotic exposure, and this requires further study to elicit whether the choice of antibiotic or duration of exposure is significant. Considering that multiple studies in NSCLC have demonstrated a stronger association of worse ICI outcomes with ABX closer to the initiation of ICI, this result may be a statistical effect due to the large sample size of the study; if it is an effect specific to melanoma, the mechanism is currently unclear.

Evidently, timing of ABX is thus an important factor that associates with poorer outcomes when given closer to commencement of ICI. The effect of choice of antibiotic and antibiotic duration is unknown.

The strength of the interaction between antibiotic administration and ICI outcomes relative to chemotherapy was shown in a study by Cortellini et al. In this multicentre European study, 950 patients with metastatic NSCLC treated with first-line pembrolizumab were compared to 595 patients treated with chemotherapy. OS was significantly worse after antibiotic administration (HR = 1.42 (95% CI 1.13 to 1.79); *p* = 0.0024) in the ICI cohort but not in the chemotherapy cohort [91]. This suggests that ABX exposure is more influential on the activity of immunotherapy than chemotherapy, which fits with our understanding of the GM’s interaction with the immune system.

More recently, a retrospective analysis of 302 patients treated with first-line combination chemo-immunotherapy for stage IV NSCLC identified that ABX prior to treatment was not associated with a difference in OS [90]. This suggests that the phenomenon may apply only to those treated with ICI alone; however, no conclusion can be made from this single study, and prospective data is needed.

It is not ethically feasible to perform a prospective randomised controlled trial of immunotherapy with and without ABX to determine whether the inferior outcomes with ABX are a cause or association and therefore the quality of evidence to answer this question will likely be limited to at best a rigorous prospective study of real-world data. Accepting this and attempting to partially account for these difficulties, a Dutch group performed a retrospective matched cohort study of 221 patients who received ICI for NSCLC matched to historical controls who received chemotherapy for NSCLC. Somewhat contradictory to the findings of Cortellini et al. [91], the magnitude of reduction in OS related to ABX exposure in patients who received chemotherapy was the same as in those who received ICI and therefore the authors argue that the dysbiosis secondary to ABX is unlikely to be the cause of worse outcomes, and that the differences seen must be due to confounding.

## The GM and ICI outcomes in other solid tumours

The literature on use of ICI in solid tumours other than NSCLC, in particular melanoma, forms much of the groundwork for hypotheses on the relationship between ICI outcomes and the microbiome. Patterns of treatment response to ICI and irAEs are reported in tumour-specific settings in general; however, tumour-agnostic effects are plausible and have been observed, in particular with certain irAEs [57]. It is likely that NSCLC is no exception to these broad effects.

### Melanoma, the GM and ICI response

In this section, we will discuss a number of studies in which certain GM profiles have been associated with response to ICI in melanoma. Metastatic melanoma was the first US Food and Drug Administration (FDA)-approved indication for the ICI ipilimumab, a monoclonal antibody against CTLA-4, and this approval was granted over a decade ago. PD-1/L1 inhibitors followed shortly afterwards, and consequently the most comprehensive evidence of antibiotic-related dysbiosis associating with negative ICI outcomes is in patients with melanoma. With cure rates and long-term responses in stage III [103] and IV [104] disease respectively increasing with ICI use in melanoma, efforts to understand the mechanisms and potentially responsible microorganisms for primary and secondary resistance to ICI are ever evolving.

Pre-clinical evidence of an interaction between the GM and the activity of anti-CTLA-4 therapy [80], as well as anti-PD-1 therapy [105], showed strong correlations between the presence of *Bacteroides* and *Bifidobacterium* and improved response to ICIs, respectively.

In a manuscript reported by Vétizou et al., the authors investigated the role of the GM in CTLA-4 inhibition for melanoma [80]. In the first instance, germ-free and ABX-treated mice were shown to have no response to the ICI in Ret-melanoma tumour models compared to controls of specific-pathogen free mice in which progression was halted by a CTLA-4 inhibitor. This strongly suggests that commensal flora play a key role in host-tumour immunity and are required for response to CTLA-4 inhibition. Simultaneously, patients with metastatic melanoma and NSCLC had peripheral blood lymphocytes tested after two doses of ipilimumab and these showed Th1 lymphocytes activated against particular *Bacteroides* species were prominent in post-ICI blood samples. When these lymphocytes were injected into germ-free and antibiotic-treated mice, a response was induced in the tumour model, implying a cross-antigenicity between *Bacteroides* and the tumour cells.

In the first prospective clinical study to link the GM to response to ICI in melanoma, Frankel et al. analysed the baseline GM via metagenomic shotgun sequencing in 39 patients treated with single and combination checkpoint inhibitors for advanced melanoma [82]. The presence of a *Bacteroides* species, *Bacteroides caccae*, was associated with higher response rates to ipilimumab, nivolumab, ipilimumab plus nivolumab, and pembrolizumab monotherapy (*p* = 0.032).

In a third melanoma manuscript, Sivan et al. showed that spontaneous antitumor immunity, as well as enhancement of tumour response to PD-L1 inhibition, could be induced by altering the GM of melanoma murine models by methods such as faecal transfer [105]. 16S rRNA sequencing identified that the presence of *Bifidobacterium* was associated with response to ICI. Oral gavage of a combination of *Bifidobacterium* species augmented tumour control in the setting of a PD-L1 antibody compared to controls, underscoring a mechanistic link.

In another translational study, Matson et al. showed that murine melanoma models had better anti-tumour response to immunotherapy after FMT from human responders to ICI [106]. Simultaneously, the stool was obtained from a group of 42 patients with metastatic melanoma treated with ICI (38 = anti-PD-1, 4 = anti-CTLA-4), and the GM was analysed using a combination of 16S rRNA sequencing and shotgun metagenomic sequencing. In this group, *Bifidobacterium longum*, *Collinsella aerofaciens* and *Enterococcus faecium* all associated with improved response rates to ICI (*p* < 0.05).

A larger study by Gopalakrishnan et al. reported the association of GM composition with outcomes in 112 patients treated with anti-PD-1 for metastatic melanoma, as measured by a combination of 16S rRNA sequencing and shotgun metagenomic sequencing. A relatively high quantity of Ruminococcaceae (*p* < 0.01) as well as higher alpha diversity of the GM (*p* < 0.01) associated with higher rates of response to ICI [107].

It is noteworthy that all of the above melanoma studies report multiple different bacteria with varying strengths of associations to response or ICI resistance. Selecting a combination of the bacteria with the strongest associations to outcomes may be the next step in refining a predictive GM profile and target for GM modifications. Early reports from a group in the UK describe a predictive signature that has been developed based on the 9 gut bacteria found to be most significantly associated with response to ICI in melanoma, with 93% accuracy for differentiating patients who respond to those who have no response to PD-1 inhibitors, CTLA-4 inhibitors or combinations of both, based on shotgun metagenomic sequencing of stool samples [108, 109]. The MELRESIST study validated this signature in four published cohorts [78, 82, 106, 107]. If this predictive tool is developed for clinical practice, then it may be possible to develop a similar tool for NSCLC.

### Melanoma, the GM and irAEs

A limited number of studies use sequencing of the GM to identify microbiota associated with irAEs in melanoma. Dubin et al. presented the first evidence that the GM may be linked to ICI-induced colitis in patients with advanced melanoma [110]. Presence of significant quantities of *Bacteroides* in the GM, identified by using a combination of 16S rRNA sequencing and shotgun metagenomic sequencing, was associated with reduced risk of ipilimumab-induced colitis in this study (*n* = 34, *p* < 0.05) and another prospective study by Chaput et al. (*n* = 26) [111]. By comparison, Chaput’s group observed that patients with gut microbiota enriched with the *Faecalibacterium* genus and other Firmicutes, identified by 16S rRNA sequencing, had a higher rate of long-term clinical benefit as well as colitis secondary to the CTLA-4 inhibitor. These patients exhibited lower circulating levels of regulatory T cells in their blood, pointing to a possible mechanistic link between response to ipilimumab being associated with toxicity in the setting of this GM profile.

In a study by Mohiuddin et al. [102] of 568 patients (*n* = 114 exposed to ABX) with stage III and IV melanoma treated with ICI, moderate to severe immune-mediated colitis was significantly more common in ABX-exposed patients, with a hazard ratio of 2.14 for developing this irAE (95% CI 1.02 to 4.52; *p* = 0.046). The authors note the possibility that, after ABX exposure, there is regrowth of pro-inflammatory bacterial species and/or inhibition of regulatory T lymphocytes in the gut which are normally induced by an intact GM. Stool sequencing was not undertaken during this study.

### Renal cell carcinoma, the GM and ICI outcomes

Renal cell carcinoma (RCC) is another ICI-responsive disease in the metastatic setting [112]. Derosa et al. have reported that *Clostridium hathewayi* [113], and recent ABX exposure, are associated with lower objective response rates to ICI in metastatic RCC (*n* = 121, *p* < 0.01) [100]. In another multicentre retrospective study of 93 patients with metastatic RCC, ABX exposure was the only factor correlating with outcomes, with lower response rates in the ABX cohort [114]. ABX exposure associated with worse OS (HR for death: 2.306; 95% CI 1.155–4.601, *p* = 0.018) vs. patients not treated with ABX. These findings are replicated in NSCLC as described below, as well as melanoma, and RCC as described above, suggesting that this is a class effect on the mechanism of ICI action and is likely tissue-agnostic.

*Akkermansia muciniphila* enrichment in stool of patients who respond to ICI is also replicated amongst at least these three tumour types. In RCC, Routy et al. found *A. muciniphila* was enriched in the stool of responders to PD-1 inhibition upon serial metagenomic shotgun sequencing of stool samples in 40 patients [78]. Salgia and colleagues replicated this finding upon testing stool of 31 patients before starting ICI for metastatic RCC (*n* = 24 nivolumab monotherapy, *n* = 7 combination nivolumab plus ipilimumab) [115]. In this group, they demonstrated that a high relative abundance of *A. muciniphila* over time was seen in patients who derived clinical benefit from ICIs. This study also observed that greater microbial alpha diversity was associated with clinical benefit from ICI in this patient cohort (*p* = 0.001).

## The GM Composition and ICI Outcomes in NSCLC

### Efficacy of ICI in NSCLC and the GM

Based on strong pre-clinical evidence and evidence from other tumours suggesting the GM contents influence the activity of ICI, multiple studies have been conducted looking at the relationship between the GM content and the outcomes of ICI in patients with NSCLC.

The first study exploring the relationship between the GM and NSCLC outcomes from ICI was published by Routy et al. in 2018 [78]. In this study of 100 patients with advanced NSCLC, shotgun metagenomic sequencing of the patients’ stool samples at baseline and multiple timepoints after commencement of ICI treatment was carried out. A higher gene count as well as a higher metagenomic species count, both of which are measures of richness of the microbiota, was associated with improved progression free survival (PFS) at 6 months by Response Evaluation Criteria in Solid Tumors (RECIST) v1.1 [116, 117] (*p* = 0.002). Further exploring the theory of GM composition influencing ICI outcomes, the contents of the GM were analysed by bacterial genus and species. The authors identified that anaerobes such as Firmicutes and *Akkermansia muciniphila* were in abundance in patients responding to ICI. *Akkermansia muciniphila* was present in 61% (34/56) of patients with clinical benefit vs. 34% (15/44) of patients with no response to ICI (*p* = 0.007). On the other hand, other bacteria were found to be more abundant in non-responders, such as *Parabacteroides*, and several species within the Clostridiales order. Hence, richness and pro-response organisms may influence ICI activity positively, and pro-resistance organisms in the GM may counteract ICI. Of note, these analyses included patients from both the ABX and no-ABX groups.

In a separate Chinese cohort of patients, a similar analysis was undertaken by prospective 16S rRNA gene sequencing of stool samples before and during therapy in 37 patients treated with the PD-1 inhibitor nivolumab for advanced NSCLC [118]. A higher GM diversity was identified in patients who demonstrated a radiologic response to treatment at 3 months vs. non-responders. In addition, improved PFS rates were seen in patients with baseline stool samples that were enriched with *Alistipes putredinis*, *Bifidobacterium longum* and *Prevotella copri*. Interestingly, *Ruminococcus_unclassified* was enriched in patients who did not respond to ICI. The difference in pro-response organisms amongst these studies is noteworthy, particularly in the case of the genus *Ruminococcus*, which appears to be associated with non-response here, but has a pro-response association in multiple other studies of ICI in solid tumours [65, 107, 119].

Exploring the same question in a Japanese population, Hakozaki et al. examined faecal samples from 70 Japanese patients treated with ICI for advanced NSCLC [119]. Bacteria enriched in all patients with a PFS over 6 months included *Ruminococcaceae UCG 13* and *Agathobacter*. *Lachnospiraceae UCG 001* was overrepresented in patients with OS > 12 months. There was no difference in alpha diversity in patients with OS > 12 months; however, patients treated with ABX prior to ICI had lower alpha diversity compared to controls. Focusing on the 47 ABX-free patients, a higher GM alpha diversity was associated with an improved OS from ICI. In this cohort, OS was longer in those enriched with *Ruminococcaceae UCG 13* on a multivariate analysis (95% CI 0.39–0.83, *p* = 0.004). These data are again different to the Chinese cohort, which may reflect the differences in the cohorts in terms of diet, host genetics and tumoral factors, and thus requires future study.

In early-stage NSCLC, Cascone et al. studied patients on a phase 2 trial of neoadjuvant combination ICI with ipilimumab and nivolumab, and measured major pathological response (MPR) as a primary endpoint [65]. In this study, a prospective exploratory analysis of the GM was carried out. This identified that *Akkermansia* species and Ruminococcaceae were in higher abundance in the GM of those patients who sustained an MPR from neoadjuvant ipilimumab plus nivolumab. This was a remarkable discovery given the findings of Routy and Hakozaki noted previously, and in particular adds weight to the case for *A. muciniphila* as a key pro-response organism.

A detailed report by Derosa et al. published in February 2022 confirms the strong links between intestinal *Akkermansia* species, in particular *A. muciniphila* and outcomes of ICI in NSCLC in pre-clinical and prospective clinical experiments [85]. The authors also explore the nuances of the relative abundance of this key pro-response organism and the associated gut microbial constituents. In a cohort of 338 patients treated with PD-1 inhibition for NSCLC (first line = 98, second line and beyond = 240), presence of *A. muciniphila* (*n* = 131) in stool was associated with a higher objective response rate (28% vs. 18%, *p* = 0.04) and longer median OS (18.8 vs. 15.4 months, HR 0.72, 95% CI 0.73–1.62, *p* = 0.03). When ABX-treated mice were administered FMT from 29 of these patients, absence of *A. muciniphila* in the FMT was associated with resistance to ICI in the murine tumour models. The presence of *A. muciniphila* in patients’ baseline stool samples was associated with enrichment of other pro-response microbiota in the GM, such as Ruminococcacae and Lachnospiracae family members, and *Alistipes* species. On the other hand, absence of *A. muciniphila* associated with the presence of detrimental organisms such as *Veillonella parvulla*, *Actinomyces* species and the *Clostridium* genus. Thus, the presence of *A. muciniphila* may result in a “collateral commensalism” which contributes to positive outcomes of ICI in NSCLC. Focusing on the group with *A. muciniphila* present, the relative richness of *A. muciniphila* was measured, and unexpectedly an overrepresentation over *A. muciniphila* was found in patients with OS < 12 months vs. those with OS > 12 months. Presence of *A. muciniphila* in lower proportions of the total gut microbiota up to 4.799% (77th percentile) was associated with higher alpha diversity and improved overall survival. Both absence of *A. muciniphila* and excessive dominance of *A. muciniphila* were associated with negative outcomes, and lower alpha diversity, emphasising the importance of alpha diversity and the contribution of other pro-response commensals.

### irAEs in NSCLC and the GM

Clinical studies reporting on the effect of the GM on irAEs in NSCLC are sparse relative to the literature on efficacy. We identified four clinical studies which measured the GM of patients who received ICI for NSCLC and report an association between the GM and irAEs. As shown already, many effects of the GM on ICI may be drug-specific rather than disease-specific, and information can be extrapolated from studies of other tumours as described above.

The most plausible direct GM-irAE relationship in terms of causality is with immune-related diarrhoea and colitis, given the localised anatomic nature of the pathology to the inciting antigen. A Chinese study of the GM of 26 patients treated with anti-PD-1 therapy (nivolumab = 16, pembrolizumab = 10) for advanced lung cancer (NSCLC = 22, small cell lung cancer = 4) sought to identify particular bacterial species as predictive of local immune reactions in the gut [120]. Upon analysis of 16S rRNA results for each patient’s faecal samples, *Bacteroides* (*p* = 0.0398), *Parabacteroides* (*p* = 0.0145) (both of Bacteroides phylum) and *Phascolarctobacterium* (*p* = 0.0090) (Firmicutes phylum) were more abundant in diarrhoea-free patients, and *Veillonella* (*p* = 0.0348) (Proteobacteria phylum) was more abundant in patients who experienced diarrhoea [120]. It is not known if the effects of these organisms are entirely local in this instance, or mediated through a systemic immune response.

The relationship between the GM and irAEs may also extend beyond GI irAEs, suggesting a systemic effect. In their study of Japanese patients, Hakozaki et al. reported differences in GM composition between patients with advanced NSCLC treated with anti-PD-1/PD-L1 who experience ≥ grade 2 irAEs of any tissue or organ and those who experienced mild (grade 1) or no irAEs [119]. The presence of pre-treatment *Akkermansia* species was associated with a lower severity of irAEs. Conversely, *Agathobacter* was associated not only with more severe irAEs but also with improved clinical outcomes. The importance of the abundance of *Akkermansia* species for positive outcomes of ICIs is possibly greater again in light of this evidence; however, it is notable that associations between irAE, efficacy and specific microbial features may be different across tumour types and patient cohorts.

A third study by Chau et al. examined the potential role of the GM in development of irAEs from ICIs [121]. In this single-centre prospective cohort study from the USA, 33 patients with advanced NSCLC were compared to 32 healthy controls from the local region. The majority of patients received pembrolizumab with a platinum-based doublet of chemotherapy (one patient received atezolizumab combined with paclitaxel). The authors analysed the gut microbiome with 16S rRNA sequencing at baseline prior to ICI treatment. The baseline GM of healthy controls demonstrated higher alpha diversity than those with NSCLC. Specific bacterial species in abundance in the GM of patients who did not experience irAEs were *Bifidobacterium* (*p* = 0.001) and *Desulfovibrio* (*p* = 0.0002).

Finally, in the NEOSTAR trial [65], no difference was identified in patient microbial features by 16S rRNA sequencing in those with $\le$ 1 treatment-related adverse events vs. those who experienced ≥ 2 treatment-related adverse events. In a cohort of 20 patients treated with nivolumab with available pre-treatment faecal samples, the GM was observed to have an abundance of *Dialister* sp. in patients with reduced toxicity to nivolumab. Similarly, *Bifidobacterium*, *Enterobacter* spp. and an unclassified genus of Erysipelotrochaceae were associated with reduced toxicity to combined nivolumab plus ipilimumab in 19 patients with available faecal specimens. *Bifidobacterium* was associated with an improved toxicity profile to an aggressive combination ICI approach.

Evidently, much more work is needed in identifying GM predictors of irAEs. These four studies have minimal overlap in terms of the organisms identified as associating with toxicity: Only *Bifidobacterium* was reported in more than one study. Ten organisms or groups of organisms have been described as anti-irAE and three have been reported as pro-irAE.

## Targeting the GM to Augment ICI Outcomes

### Microbiome-based therapeutics in other solid tumours

Microbiome-based therapies have been implemented in combination with ICI re-challenge in a bid to rescue ICI failures. In the first clinical trial of this nature to be reported, the response rate to re-challenge of nivolumab after progression of melanoma post-ICI, combined with FMT from donors with complete response post-ICI, was 30% (3 of 10 patients) in a phase 1 study of feasibility and safety [122].

Davar et al. simultaneously reported a single-arm melanoma study of fifteen patients with primary resistance to PD-1 inhibition who were given responder-derived FMT in combination with pembrolizumab [123]. Six patients (40%) derived clinical benefit: One complete response, two partial responses and three patients had stable disease for over 12 months.

As an alternative to transplantation of the entire faecal microbiota, select organisms can also be administered in high concentrations in the form of probiotics. Reports from an open-label randomised phase 1B study by Dizman et al. show [124] promising activity with the addition of the butyrate-producing bacterium *Clostridium butyricum*, the key constituent of the probiotic CBM-588, to the GM of patients with RCC. Patients were randomised in a 2:1 ratio to receive a twice-daily oral preparation of CBM-588 in combination with ipilimumab 1 mg/kg and nivolumab 3 mg/kg every 3 weeks for four cycles followed by nivolumab 480 mg every 4 weeks vs. this ICI regimen alone. The PFS butyric was 12.7 months in the CBM-588-treated group versus 2.5 months in the control arm (HR 0.15, 95% CI 0.05–0.47, *P* = 0.001), suggesting that this strategy is worthy of further study in phase 2 and 3 settings. Median OS has not been reached in either group at 12.2-month median follow-up.

Microbiome-based therapeutics have been explored as potential treatments for irAEs, following the example of *C. difficile* colitis which can be treated by targeting dysbiosis with faecal microbiota transplantation (FMT) from healthy individuals [125]. There are anecdotally successful reports of the use of faecal microbiota transplant (FMT) via colonoscopy from healthy individuals to two patients with refractory ICI-induced colitis after failure of steroids, infliximab and vedolizumab in the setting of urothelial cancer and prostate cancer [126]. A separate case report of non-response of ICI-related colitis to unselected donor FMT, which was quickly followed by rapid progression of the patient’s metastatic melanoma, demonstrates the caveat of this approach [127] and the need for randomised controlled clinical trials.

### The GM as a therapeutic target in patients with NSCLC on ICI

As noted above, there are measurable differences between the GM of patients with NSCLC who respond to ICI and non-responders, and those who experience clinically significant irAEs vs. non-significant or no irAEs. This led to pre-clinical testing of manipulation of the GM via methods such as probiotic administration, as well as FMT. In Routy’s report [78], the stool of 8 patients with NSCLC, half of which were responders to ICI, was used for FMT in mice with solid tumour models, and showed improved response to ICI in the mice which received FMT from human responders. An oral gavage with *A. muciniphila* and *Enterococcus hirae* probiotics resulted in reversal of the compromised efficacy of ICI in a lung cancer model in mice.

Studies of FMT in humans with NSCLC are not reported to date and theoretical benefits are extrapolated from studies of other tumours. A randomised prospective trial of FMT in addition to second-line nivolumab for advanced NSCLC is ongoing in Europe (ClinicalTrials.gov Identifier: NCT04924374).

A retrospective review of 118 Japanese patients who received ICI for advanced NSCLC found that 39 patients (33%) received the probiotic CBM-588 within 6 months prior to commencement of ICI and/or concurrently with ICI, for indications such as post-antibiotic gastrointestinal symptoms. The cohort who received CBM-588 had a significantly longer overall survival, with a hazard ratio for death of 0.27 (95% CI, 0.11–0.66) in a multivariate analysis accounting for factors including age, ECOG performance status, histology, PD-L1 status, initial stage and ICI therapy line. These results are impressive but prospective randomised data are needed.

No reports of manipulation of the GM in NSCLC for treatment of irAEs were found at the time of this review.

## Discussion

The relationship between the GM and both the adaptive and innate immune system is bidirectional. It is likely that a certain GM profile is favourable for an individual’s health, and dysbiosis may refer to anything outside of this, in terms of both the presence of favourable microbiota and the diversity of the GM. Modern gene sequencing techniques are contributing to efforts to identify to the optimal GM for response to systemic therapy in early and late-stage cancer, in particular for ICIs. Building on the exceptional progress made by ICIs in NSCLC is the goal of these efforts, with a focus on resistance to ICI as well as reducing harm from irAEs. The latter will be further scrutinised as ICI is moved forward to the early stages of disease. The mechanism for the influence of the GM on ICI outcomes is complex and poorly understood: Continuous exposure of immune cells at the intestinal border to non-pathogenic microbial antigens apparently primes the immune system to the infiltration of pro-inflammatory cytokines as well as helper and cytotoxic T lymphocytes into the tumour microenvironment. This process is amplified upon introduction of ICIs. ABX appear detrimental to outcomes of patients treated with ICI for NSCLC, and the strength of association is likely inversely related to the time between exposure to ABX and initiation of ICI. Individual organisms associated with positive outcomes can vary between studies and tumour types, and this may be explained in part by work on the pro-response organism *A. muciniphila* in NSCLC which shows that alpha diversity and healthy collateral commensalism are co-factors required for improved response rates and survival outcomes. Pre-clinical evidence strongly suggests modulation of the GM can be a successful therapeutic adjunct to ICI, and limited clinical studies in melanoma support this. Moreover, the rescue of patients from treatment-refractory irAEs such as colitis with FMT is an attractive theory with a paucity of clinical data at this time.

Prospective, randomised data in a population of patients with NSCLC is awaited to prove the concept of dysbiosis affecting ICI outcomes, and, in order to change practice, testing of the GM will need to be standardised, validated and logistically feasible to incorporate into routine care. A major barrier to breakthroughs remains the complexity of the mechanism of interactions between the GM, NSCLC, ICI and drugs such as ABX. This complexity is evidenced by the sometimes-contradictory links between certain bacteria and positive or negative outcomes**.** In the absence of ABX, multiple studies show GM composition strongly correlates with outcomes, and this is supportive of the dysbiosis theory. The variation in reporting of the GM composition includes describing organisms at a range of levels in the taxonomic ranks. In order to strengthen evidence going forward, a uniform approach to measurement and reporting of the composition of the GM in patients on ICI is warranted, and a computative approach has been assessed in a mixed cohort of patients with solid tumours treated with ICI, which appears to be strongly predictive of non-response [128], but requires prospective validation. The modulation of the GM by diet, FMT or probiotic administration could theoretically augment clinical activity of ICI in NSCLC; however, FMT remains a last-line largely experimental approach for treatment-refractory checkpoint inhibitor colitis at the time of writing. Study of FMT from responding patients to non-responding patients is confounded by the unpredictable nature of ICI responses; for example, in the single-arm study of FMT combined with re-challenge of nivolumab in patients with melanoma [122], the response rates were similar to historic controls in the literature of patients with melanoma re-challenged with ICI after progression [129], and therefore it is unclear what role the FMT had in the benefit derived by these patients from ICI re-challenge. Indications for ICI within NSCLC and in general are expanding, and more widespread use should lead to the possibility for recruitment to randomised trials of probiotics and/or FMT vs. standard of care for indications such as treatment-refractory immune-mediated colitis as well as primary and secondary resistance to ICI.

The scope and content of this non-systematic review are limited by the quality of the evidence available on the topic; for example, evidence of causality of ABX affecting ICI outcomes is not sufficient at this point to make a recommendation on changing antibiotic prescription practice. Due to the rapidly evolving nature of this field, it is impossible to give a comprehensive account of the published literature, and included information may be biased according to the authors’ pre-existing knowledge and clinical experience; however, every effort was made to include the most up-to-date information in a focused but detailed manner with a true representation of the primary literature.

In conclusion, the pre-clinical evidence for a significant effect of the GM composition on ICI outcomes shows a mechanism for how the GM can mobilise the immune system against cancer, and there is compelling clinical evidence from retrospective and exploratory analyses that ABX and antibiotic-induced dysbiosis in particular have a deleterious effect on outcomes in NSCLC treated with ICI, beyond that accounted for by baseline clinical features of frailty or disease biology that underlie the indication for antibiotic treatment. ABX will remain necessary for intercurrent infection, and judicious use is needed in general. It is premature at this time to recommend a particular probiotic to augment the anti-cancer effect of ICI or to reduce the incidence/severity of IRAEs, outside of a clinical trial. With emerging methods to standardise measurement of the microbiome and clinical trials of therapeutics targeting the microbiome already in progress, the next decade may well see measurement and alteration of the GM for patients undergoing ICI for NSCLC as a key component of routine clinical care to both maximise response and minimise toxicity.
