# Correction to: The gut microbiome, immune check point inhibition and immune‑related adverse events in non‑small cell lung cancer

**DOI:** 10.1007/s10555-022-10062-2

**Published:** 2022-08-19

**Authors:** Philip Bredin, Jarushka Naidoo

**Affiliations:** 1 Beaumont RCSI Cancer Centre, Dublin, Ireland; 2 The Bloomberg-Kimmel Institute of Cancer Immunotherapy, Johns Hopkins University School of Medicine, Baltimore, MD USA; 3 Department of Oncology, Johns Hopkins University School of Medicine, Baltimore, MD USA

**Correction to: Cancer and Metastasis Reviews (2021) 41:347–366**

**https://doi.org/10.1007/s10555-022-10039-1**

The article The gut microbiome, immune check point inhibition and immune‑related adverse events in non‑small cell lung cancer, written by Philip Bredin and Jarushka Naidoo, was originally published online first without Open Access. After publication in volume 41, issue 2, page 347–366, the author decided to opt for Open Choice and to make the article an Open Access publication. Therefore, the copyright of the article has been changed to © The Author(s) 2022, and the article is forthwith distributed under the terms of the Creative Commons Attribution 4.0 International License, which permits use, sharing, adaptation, distribution and reproduction in any medium or format, as long as you give appropriate credit to the original author(s) and the source, provide a link to the Creative Commons licence and indicate if changes were made. The images or other third party material in this article are included in the article’s Creative Commons licence, unless indicated otherwise in a credit line to the material. If material is not included in the article’s Creative Commons licence and your intended use is not permitted by statutory regulation or exceeds the permitted use, you will need to obtain permission directly from the copyright holder. To view a copy of this licence, visit http://creativecommons.org/licenses/by/4.0. Open Access funding enabled and organized by Projekt DEAL.

The original article has been corrected.

